# Supplementary material for: High-resolution detection of copy number alterations in single cells with HiScanner
Source: Nat Commun. 2025 Jul 1;16:5477. doi: 10.1038/s41467-025-60446-5 (PMC12214996; doi:10.1038/s41467-025-60446-5)
Supplement: Supplementary file 2 — Description of Additional Supplementary Files [file 41467_2025_60446_MOESM2_ESM.pdf]

Description of Additional Supplementary Files

Supplementary Data 1: BED files containing genomic coordinates of simulated CNAs
