# Supplementary material for: High-resolution detection of copy number alterations in single cells with HiScanner
Source: Nat Commun. 2025 Jul 1;16:5477. doi: 10.1038/s41467-025-60446-5 (PMC12214996; doi:10.1038/s41467-025-60446-5)
Supplement: Supplementary file 5 — Reporting Summary [file 41467_2025_60446_MOESM5_ESM.pdf]

nature portfolio

Corresponding author(s): Peter J. Park, Mark D. Johnson

Last updated by author(s): Mar 28, 2025

Reporting Summary

Nature Portfolio wishes to improve the reproducibility of the work that we publish. This form provides structure for consistency and transparency in reporting. For further information on Nature Portfolio policies, see our Editorial Policies and the Editorial Policy Checklist.

Statistics

For all statistical analyses, confirm that the following items are present in the figure legend, table legend, main text, or Methods section.

n/a	Confirmed	
☐	☒	The exact sample size (*n*) for each experimental group/condition, given as a discrete number and unit of measurement
☐	☒	A statement on whether measurements were taken from distinct samples or whether the same sample was measured repeatedly
☐	☒	The statistical test(s) used AND whether they are one- or two-sided<br>*Only common tests should be described solely by name; describe more complex techniques in the Methods section.*
☐	☒	A description of all covariates tested
☐	☒	A description of any assumptions or corrections, such as tests of normality and adjustment for multiple comparisons
☐	☒	A full description of the statistical parameters including central tendency (e.g. means) or other basic estimates (e.g. regression coefficient) AND variation (e.g. standard deviation) or associated estimates of uncertainty (e.g. confidence intervals)
☐	☒	For null hypothesis testing, the test statistic (e.g. *F*, *t*, *r*) with confidence intervals, effect sizes, degrees of freedom and *P* value noted<br>*Give P values as exact values whenever suitable.*
☒	☐	For Bayesian analysis, information on the choice of priors and Markov chain Monte Carlo settings
☒	☐	For hierarchical and complex designs, identification of the appropriate level for tests and full reporting of outcomes
☒	☐	Estimates of effect sizes (e.g. Cohen's *d*, Pearson's *r*), indicating how they were calculated

Our web collection on statistics for biologists contains articles on many of the points above.

Software and code

Policy information about availability of computer code

Data collection	No data collection was carried out for our previously generated PTA-amplified single neuron and oligodendrocytes and matched bulk WGS. The meningioma data were downloaded from the sequencing facility.
Data analysis	Data were analyzed in a conda environment with more than 50 python packages. We provide the analysis code as well as detailed instructions about environment set up and software installation at https://github.com/parklab/hiscanner.

For manuscripts utilizing custom algorithms or software that are central to the research but not yet described in published literature, software must be made available to editors and reviewers. We strongly encourage code deposition in a community repository (e.g. GitHub). See the Nature Portfolio guidelines for submitting code & software for further information.

Data

Policy information about availability of data

All manuscripts must include a data availability statement. This statement should provide the following information, where applicable:

- Accession codes, unique identifiers, or web links for publicly available datasets
- A description of any restrictions on data availability
- For clinical datasets or third party data, please ensure that the statement adheres to our policy

Newly generated scWGS data for 179 PTA-amplified single-cells and two matched tumor/blood bulk WGS of the initial and recurrent meningioma samples are accessible at European Genome-Phenome Archive under dataset ID EGAD50000001254. Previously generated scWGS and matched bulk WGS of PTA neurons are

available for download at dbGaP, accession number phs001485.v3.p1. scWGS of MDA and PTA oligodendrocytes are available for download at NIAGADs with accession number NG00162.

Research involving human participants, their data, or biological material

Policy information about studies with human participants or human data. See also policy information about sex, gender (identity/presentation), and sexual orientation and race, ethnicity and racism.

Reporting on sex and gender	The meningioma matched tumor and blood samples were obtained from a male patient. Sex/gender-based analyses were not performed as this is a single-patient case study.
Reporting on race, ethnicity, or other socially relevant groupings	Not relevant to our study, because the nature of this study is to investigate the cellular composition and clonal evolution within a single tumor.
Population characteristics	BO5-E and BO5-5 are grade II and grade Ill tumors, respectively, collected from the same patient during two surgical operations. The tumor grade was determined by a board-certified pathologist.
Recruitment	The single male subject was recruited through the Brigham and Women's Hospital neurosurgery department. The patient underwent two surgeries: first for an initial grade II meningioma (B05-E) and later for its recurrence as a grade III tumor (B05-5). Written informed consent was obtained from the patient, allowing for the collection and analysis of the tumor and blood samples. The recruitment and consent process was conducted in accordance with the human subjects institutional review board protocol approved by the Brigham and Women's Hospital. This case was selected for study because the availability of matched initial and recurrent meningioma samples from the same patient is rare, providing a unique opportunity to study tumor evolution as a detailed case study.
Ethics oversight	The study is conducted under the auspices of a human subjects institutional review board protocol approved by the Brigham and Women's Hospital.

Note that full information on the approval of the study protocol must also be provided in the manuscript.

Field-specific reporting

Please select the one below that is the best fit for your research. If you are not sure, read the appropriate sections before making your selection.

- [x] Life sciences
- [ ] Behavioural & social sciences
- [ ] Ecological, evolutionary & environmental sciences

For a reference copy of the document with all sections, see nature.com/documents/nr-reporting-summary-flat.pdf

Life sciences study design

All studies must disclose on these points even when the disclosure is negative.

Sample size	No sample size calculation was performed. The number of cells collected from initial (n=88) and recurrent (n=91) meningioma was l determined based on the number of wells available in the 96-well plate. The number of PTA neurons and oligodendrocytes analyzed was determined by the number of cells available in prior studies (Luquette et al. 2022 Nature Genetics, Ganz et al. 2024 Cell).
Data exclusions	No data were excluded from the analysis.
Replication	We consider single cells from the same individual as biological replicates. Our analysis is done with computational frameworks. The codes and related algorithms are available, and we tested the reproducibility of the results by running the code in multiple times.
Randomization	Not relevant to our study because the study design focused on characterizing genomic features in existing biological samples rather than testing interventions that would require random assignment to control for potential confounding variables.
Blinding	Blinding is not relevant to our study because we performed computational analyses of genomic data using automated algorithms that process all samples identically, regardless of their biological origin.

Reporting for specific materials, systems and methods

We require information from authors about some types of materials, experimental systems and methods used in many studies. Here, indicate whether each material, system or method listed is relevant to your study. If you are not sure if a list item applies to your research, read the appropriate section before selecting a response.

Materials & experimental systems

n/a	Involved in the study
☒	☐ Antibodies
☒	☐ Eukaryotic cell lines
☒	☐ Palaeontology and archaeology
☒	☐ Animals and other organisms
☒	☐ Clinical data
☒	☐ Dual use research of concern
☒	☐ Plants

Methods

n/a	Involved in the study
☒	☐ ChIP-seq
☒	☐ Flow cytometry
☒	☐ MRI-based neuroimaging

Plants

Seed stocks	*Report on the source of all seed stocks or other plant material used. If applicable, state the seed stock centre and catalogue number. If plant specimens were collected from the field, describe the collection location, date and sampling procedures.*
Novel plant genotypes	*Describe the methods by which all novel plant genotypes were produced. This includes those generated by transgenic approaches, gene editing, chemical/radiation-based mutagenesis and hybridization. For transgenic lines, describe the transformation method, the number of independent lines analyzed and the generation upon which experiments were performed. For gene-edited lines, describe the editor used, the endogenous sequence targeted for editing, the targeting guide RNA sequence (if applicable) and how the editor was applied.*
Authentication	*Describe any authentication procedures for each seed stock used or novel genotype generated. Describe any experiments used to assess the effect of a mutation and, where applicable, how potential secondary effects (e.g. second site T-DNA insertions, mosiacism, off-target gene editing) were examined.*
